# microRNA Temporal-Specific Expression Profiles Reveal longissimus dorsi Muscle Development in Tianzhu White Yak

**DOI:** 10.3390/ijms251810151

**Published:** 2024-09-21

**Authors:** Bingang Shi, Chune Zhu, Xiangyan Wang, Youpeng Qi, Jiang Hu, Xiu Liu, Jiqing Wang, Zhiyun Hao, Zhidong Zhao, Xiaolan Zhang

**Affiliations:** Gansu Key Laboratory of Herbivorous Animal Biotechnology, College of Animal Science and Technology, Gansu Agricultural University, Lanzhou 730070, China; shibg@gsau.edu.cn (B.S.); 18394174334@163.com (C.Z.); wxy9242022@163.com (X.W.); qiyp_gsau@163.com (Y.Q.); liuxiu@gsau.edu.cn (X.L.); wangjq@gsau.edu.cn (J.W.); haozy@gsau.edu.cn (Z.H.); zhaozd@gsau.edu.cn (Z.Z.); zhangxl@gsau.edu.cn (X.Z.)

**Keywords:** longissimus dorsi muscle, intramuscular fat, miRNAs, yak

## Abstract

As a class of regulatory factors, microRNAs (miRNAs) play an important role in regulating normal muscle development and fat deposition. Muscle and adipose tissues, as major components of the animal organism, are also economically important traits in livestock production. However, the effect of miRNA expression profiles on the development of muscle and adipose tissues in yak is currently unknown. In this study, we performed RNA sequencing (RNA-Seq) on Tianzhu white yak longissimus dorsi muscle tissue obtained from calves (6 months of age, M6, *n* = 6) and young (30 months of age, M30, *n* = 6) and adult yak (54 months of age, M54, *n* = 6) to identify which miRNAs are differentially expressed and to investigate their temporal expression profiles, establishing a regulatory network of miRNAs associated with the development of muscle and adipose. The results showed that 1191 miRNAs and 22061 mRNAs were screened across the three stages, of which the numbers of differentially expressed miRNAs (DE miRNAs) and differentially expressed mRNAs (DE mRNAs) were 225 and 450, respectively. The expression levels of the nine DE miRNAs were confirmed using a reverse transcription quantitative PCR (RT-qPCR) assay, and the trend of the assay results was generally consistent with the trend of the transcriptome profiles. Based on the expression trend, DE miRNAs were categorized into eight different expression patterns. Regarding the expression of DE miRNAs in sub-trends Profile 1 and Profile 2 (*p* < 0.05), the gene expression patterns were upregulated (87 DE miRNAs). Gene ontology (GO) and Kyoto Encyclopedia of Genes Genomes (KEGG) analyses showed that the identified DE miRNAs and DE mRNAs were enriched in pathway entries associated with muscle and intramuscular fat (IMF) growth and development. On this basis, we constructed a DE miRNA–mRNA interaction network. We found that some DE mRNAs of interest overlapped with miRNA target genes, such as *ACSL3*, *FOXO3*, *FBXO30*, *FGFBP4*, *TSKU*, *MYH10* (muscle development), *ACOX1*, *FADS2*, *EIF4E2*, *SCD1*, *EL0VL5*, and *ACACB* (intramuscular fat deposition). These results provide a valuable resource for further studies on the molecular mechanisms of muscle tissue development in yak and also lay a foundation for investigating the interactions between genes and miRNAs.

## 1. Introduction

Yak (*Bos grunniens*) are endemic to the plateau region and are mainly found on the Qinghai–Tibetan Plateau and its neighboring alpine mountainous regions in China (average altitude of 3000 m); they are capable of adapting to harsh environments, such as severe cold and low oxygen. Male yak usually reach sexual maturity at the age of 2 and can be used for breeding. They provide local herdsmen with meat, milk, plush, leather, and other necessities of life and production and are an important pillar of local livestock development [1]. China is the birthplace of the yak, with about 15 million yak, accounting for 95% of the world’s total yak population [2]. Yak meat is characterized by a high protein content, a low fat content, a high mineral content, and rich nutritional value, which makes it very popular among consumers [3]. The Tianzhu white yak has better meat quality, rich nutrition, and a minimal intramuscular fat (IMF) content; however, its growth is slow and its meat yield is low [4,5,6]. Due to the limitation of geographic distribution, there are few studies on the molecular mechanisms related to fat deposition. Therefore, investigating the molecular mechanisms of muscle and fat development in Tianzhu yak is essential for the development and utilization of this breed.

Mammalian muscle tissue can be classified into three types, namely, smooth muscle, cardiac muscle, and skeletal muscle, based on differences in morphological structure and functional characteristics. Skeletal muscle, as the largest tissue in the human body, plays an important role in maintaining the body’s movement and energy metabolism, among other things [7]. Skeletal muscle development mainly involves myogenesis, adipogenesis, and myofibrillogenesis [8,9]. Several reports have indicated that muscle growth and development are regulated by many genes, transcription factors, signaling pathways, and other regulators and that skeletal muscle growth and development directly affects muscle production in livestock [10].

Fat is one of the most important components of beef and is closely related to beef quality. IMF content is the amount of fat deposited in the muscle, which affects the flavor and sensory characteristics of livestock and poultry meat, such as tenderness, juiciness, and unique flavor [11,12]. At the species level, IMF deposition is formed by the balanced action between hepatic ab initio synthesis, lipoprotein lipase, and fat intake, and the level of lipid metabolism inside and outside adipocytes also affects fat accumulation during IMF formation [11,13]. It has been shown that the differences in IMF content in porcine longissimus thoracis and semitendinosus are mainly caused by the expression of key genes for lipogenesis, glucose consumption, and fatty acid composition during fat differentiation [14]. Therefore, obtaining functional candidate genes for IMF deposition in yak is of great theoretical and practical significance. The deposition process of IMF is complex and can be regulated by a variety of genes, including mRNA, miRNA, and lncRNA.

MicroRNAs (miRNAs) are 20–23 bp endogenous non-coding regulatory RNAs in eukaryotes that inhibit translation or induce degradation of mRNAs encoding proteins containing sequences complementary to miRNAs [15,16,17]. MiRNAs play important roles in early development, cell proliferation and differentiation, apoptosis, inhibition of oncogene expression, and lipid metabolism in animals. It was reported that miR-21, miR-143, and miR-17 promoted pre-adipocyte differentiation in pigs, whereas miR-145 inhibited pre-adipocyte differentiation in pigs [18,19]. MiRNA-133 controls cardiac and skeletal muscle development in sheep [20]. MiR-14 and miR-278 in fly liposomes regulate lipid metabolism [21]. The Bta-miR-330-*SESN3*-Akt-mTOR axis plays an important role in bovine adipogenesis of intramuscular pre-adipocytes [22]. Inhibition of miR-24-3p by RT-qPCR resulted in a significant increase in the bovine target gene *CAMK2B* gene, which regulates muscle development [23]. MiR-27a-5p increases steer fat deposition partly by targeting calcium-sensing receptors (CASRs) [24]. However, there is no comprehensive understanding of how miRNAs regulate the mechanisms of yak growth and development.

In this study, we investigated the specificity of muscle tissues of Tianzhu white yak at different developmental stages using staining techniques on muscle tissues from 6-month-age, 30-month-age, and 54-month-age yak. Using RNA-Seq and RT-qPCR techniques, we determined the dynamic expression profiles of miRNAs and established the miRNA regulatory networks associated with muscle and adipose tissue development. Our aim was to understand the molecular mechanisms that regulate skeletal muscle development and fat deposition in yak and to explore miRNAs with important roles, laying the foundation for further studies on the interactions among miRNAs, mRNAs, and signaling pathways.

## 2. Results

### 2.1. Intramuscular Fat Contents of the longissimus dorsi Muscle in Yak of Different Ages

**Figure 1:**
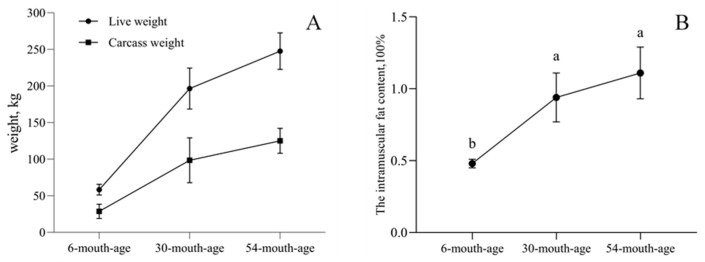
The dynamics of the live weight (**A**) and the intramuscular fat (IMF) content (**B**) across 6, 30, and 54 months of age. Different letters (a, b) indicate significant differences between different growth stages (*p* < 0.05).

The muscle and intramuscular fat (IMF) content of the longissimus dorsi muscle increased along with the development of the yak from 6 months of age to 54 months of age. Compared with the IMF content in the 6-month-age yak, those in the 30- and 54-month-age yak were significantly higher (*p* < 0.05), and the 30-month-age group also showed the fastest longissimus dorsi muscle fat deposition of the yak. However, the IMF content increased slightly from 30 months of age to 54 months of age. Among different ages, live weight and carcass weight also exhibited similar trends of change (Figure 1A,B).

### 2.2. Characterization of the longissimus dorsi Muscle Tissue at Different Ages

**Figure 2:**
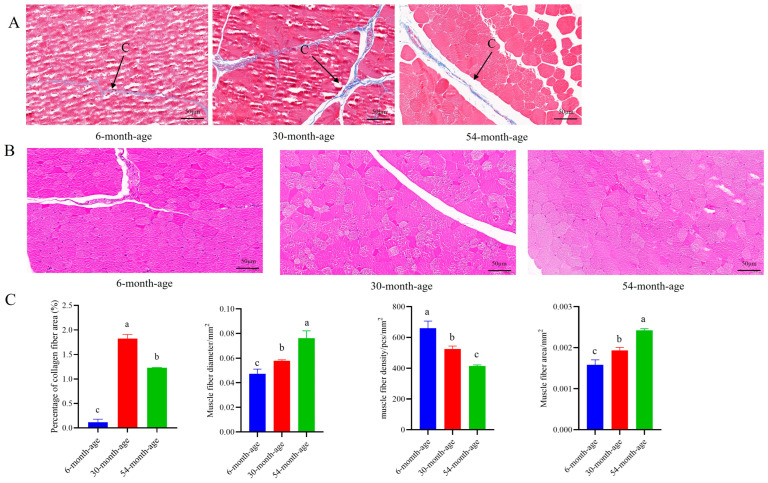
Collagen fiber staining and HE staining of Tianzhu white yak muscle tissue. (**A**) C represents collagen fibers. (**B**) Red represents a single muscle fiber. (**C**) Different lowercase letters indicate significant differences (*p* < 0.05).

Skeletal muscle consists mainly of myofibers, adipose tissue, and connective tissue, of which the connective tissue content significantly affects muscle tenderness, and its main component is collagen fibers. The collagen fibers in muscle tissue can be stained blue and myofibers red using Masson staining (Figure 2A). The muscle fibers were stained using HE staining (Figure 2B). The results are shown in Figure 2C. The collagen fiber content in the muscle tissue of 6-month-age Tianzhu white yak was the lowest, the collagen fiber content in the muscle tissue of 30-month-age Tianzhu white yak was the highest, and there was a significant difference between the three ages (*p* < 0.05). Myofiber characteristics of Tianzhu white yak differed significantly at different developmental stages. Among them, myofiber diameter and myofiber area tended to increase significantly with age and were smallest at 6 months of age and largest at 54 months of age (*p* < 0.05). Myofiber density was largest at 6 months and smallest at 54 months, and there was a significant decreasing trend (*p* < 0.05).

### 2.3. Small RNA Library Sequencing and Sequence Analysis

**Figure 3:**
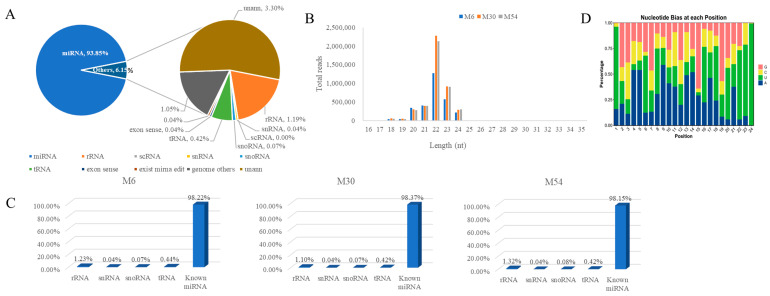
The type of identification and characterization analysis of small RNA. (**A**) Statistical analysis of the types of small RNA fragments after comparison with the database, including miRNA (existing miRNAs, known miRNAs, and novel miRNAs), rRNA, scRNA, snRNA, snoRNA, tRNA, exon sense, miRNA editing, other genome, and unann. (**B**) Statistical analysis of small RNA fragment size. (**C**) The type and percentage of small RNA in M6, M30, and M54 longissimus dorsi muscle tissues. (**D**) miRNA base preference in M6, M30, and M54 longissimus dorsi muscle tissues.

We constructed 18 small RNA libraries from the longissimus dorsi muscle tissues of yak aged 0.5 (M6), 2.5 (M30), and 4.5 years (M54). After high-throughput sequencing, the average clean reads were 13,786,039, 16,023,867, and 16,575,445 at the age stages of M6, M30, and M54, respectively. The average clean tags obtained after quality control were 13,227,222 (96.20%), 15,627,938 (96.70%), and 16,518,724 (96.47%) for subsequent analysis (Table 1). The comparison between pristine reads and the yak’s reference genome revealed a comparison rate of over 75% for each sample (Table 2). After comparison with GenBank, Rfam, and the reference, 93.85% of clean reads were identified as miRNA, and the remaining reads included rRNA, scRNA, snRNA, snoRNA, tRNA, miRNA editing, other genome and unann. (Figure 3A, Table S1). By analyzing the length characteristics of miRNAs, the length of the smallest RNAs was in the range of 20–24 nt. The miRNAs of 22 nt were the longest, followed by miRNAs with lengths of 23 nt, 21 nt, 20 nt, and 24 nt (Figure 3B). Statistics for all types of RNA sequences revealed that known miRNAs accounted for 98.22%, 98.37%, and 98.15% of the longissimus dorsi muscle tissues of the Tianzhu white yak in M6, M30, and M54, respectively (Figure 3C). In addition, the Dicer enzyme has a strong bias for the first base at the 5′ end towards the U base when recognizing and cleaving precursor miRNAs. Therefore, we analyzed the base bias of the obtained pre-existing miRNAs, and the statistical graphs of the first-base bias and the base bias of tag sites of different lengths of pre-existing miRNA tag sequences are shown below, along with the highest proportion of U base content, which accounted for 29.73% of all bases (Figure 3D).

### 2.4. Analysis of Differentially Expressed miRNAs

**Figure 4:**
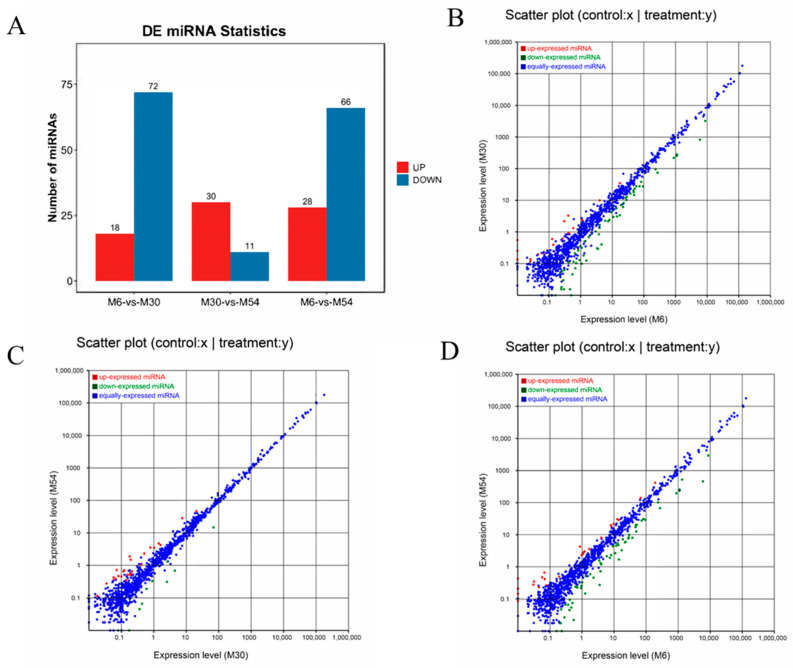
Statistical analysis of DE miRNAs. Note: Scatter plots show up- and downregulated DE miRNAs between groups, with the horizontal coordinates showing the expression levels in the control group and the vertical coordinates showing the expression levels in the test group. Red color indicates upregulated miRNAs, green color indicates downregulated miRNAs, and blue color indicates co-expressed miRNAs. (**A**) Number of up- and downregulated DE miRNAs in 3 groups of Tianzhu white yak. (**B**) Scatter plot of DE miRNAs relative to M6 at M30. (**C**) Scatter plot of DE miRNAs relative to M30 at M54. (**D**) Scatter plot of DE miRNAs relative to M6 at M54.

The screening results of DE miRNAs between groups are shown in Figure 4A (Table S2). A total of 90 DE miRNAs (18 upregulated, 72 downregulated) were identified in the M30 group relative to M6 (Figure 4A,B); a total of 41 DE miRNAs (30 upregulated, 11 downregulated) were identified in the M54 group relative to M30 (Figure 4A,C); and a total of 94 DE miRNAs (28 upregulated, 66 downregulated) were identified in the M54 group relative to M6 (Figure 4A,D).

### 2.5. Target Gene Prediction of DE miRNAs

**Figure 5:**
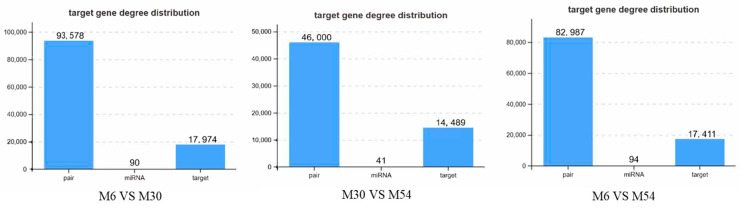
Target gene prediction for small RNA.

A total of 90 DE miRNAs were identified in the M30 group relative to M6, and 17,974 target genes could be obtained by prediction; a total of 41 DE miRNAs were identified in the M54 group relative to M30, and 14,489 target genes could be obtained by prediction; and a total of 94 DE miRNAs were identified in the M54 group relative to M6, and 17,411 target genes could be obtained by prediction (Figure 5). The intersections of the predicted results were taken as the target genes of miRNAs for the subsequent analyses.

### 2.6. STEM Analysis of the DE miRNA Expression Profiles

**Figure 6:**
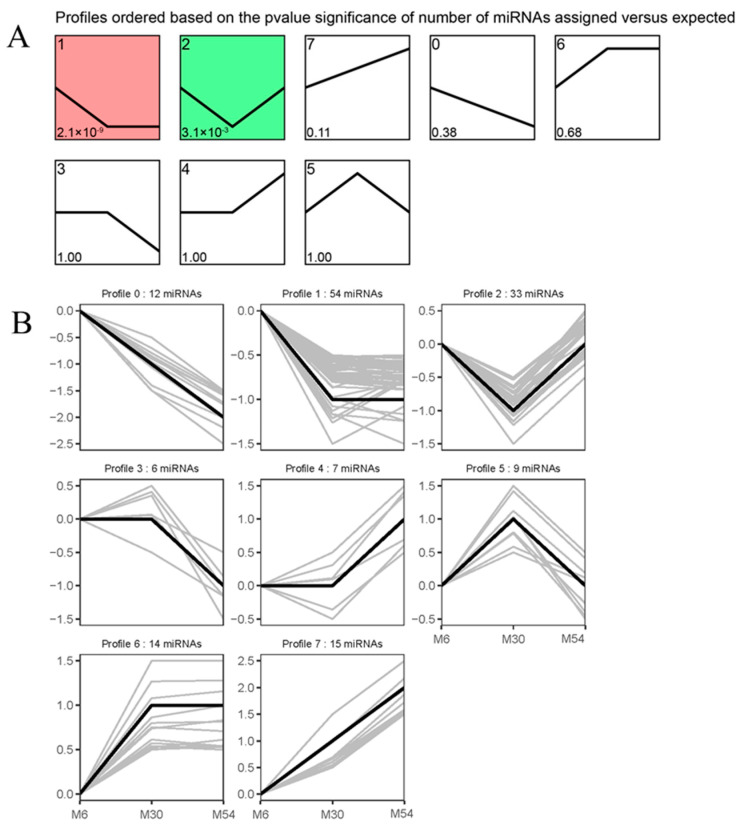
Short Time-series Expression Miner (STEM) analysis of DE miRNA expression profiles in the longissimus dorsi muscle of Tianzhu white yak. (**A**) The profile number and the number of DE miRNAs are shown in each square. The number of DE miRNAs assigned is used to order the profiles. The profiles with color (*p* < 0.05) indicate a significant enrichment trend; the profiles without color indicate a non-significant enrichment trend. (**B**) Two upregulated significant clusters of DE miRNA profiles across all three stages of development, including Profiles 1 and 2. The *X*-axis indicates the yak development state (M6, M30, and M54); the *Y*-axis shows expression changes. Black lines represent trend lines; gray lines represent the genes.

To determine temporal gene expression patterns, we used the STEM program to categorize the 159 DE miRNAs that were differentially expressed in M6 versus M30, M30 versus M54, and M6 versus M54 into eight possible expression profiles; the two expression patterns differed significantly during longissimus dorsi muscle tissue development (*p* < 0.05; Figure 6A). The integration of DE miRNAs contained in sub-trends Profile 1 and Profile 2 is noted as class-up (87 DE miRNAs) (*p* < 0.05; Figure 6B).

### 2.7. GO and KEGG Enrichment Analysis of Differentially Expressed miRNAs

**Figure 7:**
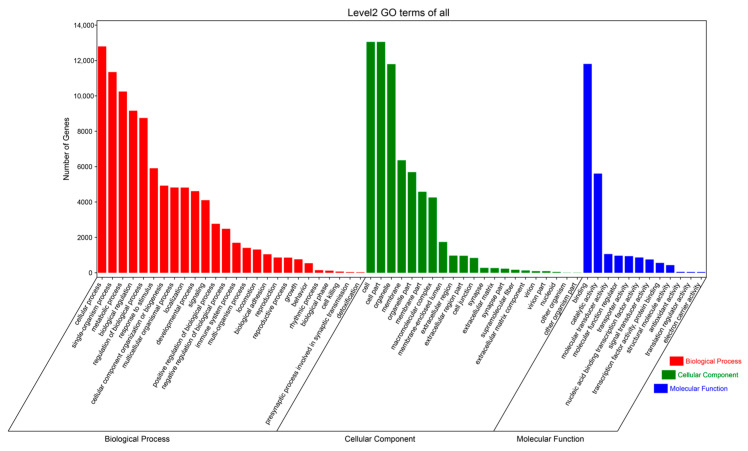
GO enrichment analysis of target genes of DE miRNAs.

**Figure 8:**
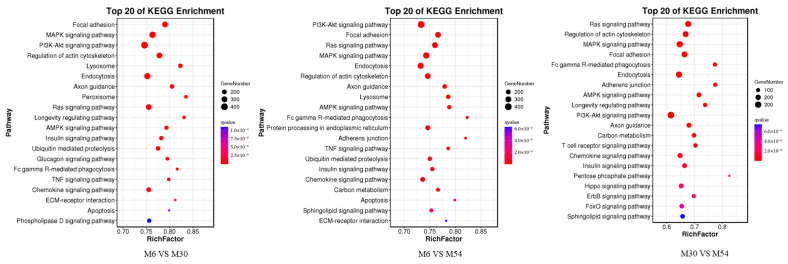
Top 20 KEGG signaling pathways enriched by DE miRNA target genes.

In order to further clarify the biological functions played by DE miRNAs, the predicted target genes were functionally annotated and analyzed by the DAVID online tool, and the annotation results for the three groups are shown in Figure 7 (Table S3). The Gene ontology (GO) annotation analysis revealed that the target genes were mainly involved in cellular (GO: 00055623), organelle (GO: 00443226), intracellular metabolism (GO: 0044237), acyl coenzyme metabolism (GO: 0006637), lipid regulation (GO: 0010883), coenzyme A biosynthesis (GO: 0015937), negative regulation of fatty acid metabolism (GO: 0045922), and other processes, exerting CoA carboxylase activity (GO: 0016421), ligase activity, formation of carbon–nitrogen bonds (GO: 0016879), purine ribonucleoside binding (GO: 0032550), cation binding (GO: 0043169), and catalytic roles (GO: 00033824). Notably, a large number of miRNA target genes are involved in processes such as metabolism and catalysis, and muscle development and fat deposition cannot be separated from catalytic metabolic processes and lipid regulation; thus, it can be seen that the screened key GO pathways play important roles in muscle tissue development. The top 20 Kyoto Encyclopedia of Genes Genomes (KEGG) terms enriched in the three combinations based on the Q-values of signaling pathways, enrichment factors, and gene numbers are shown in Figure 8 (Table S4). The major enriched signaling pathways were the fatty acid metabolism pathway (ko01212), the PPAR signaling pathway (ko03320), actin cytoskeleton regulation (ko04810), the insulin signaling pathway (ko04910), the PI3K-Akt signaling pathway (ko04151), the MAPK signaling pathway (ko04010), the AMPK signaling pathway (ko04152), the glucagon signaling pathway (ko04922), and the adipocytokine signaling pathway (ko04920). Most of the DE miRNA target genes with major enrichment in growth and development and lipid metabolism include signaling pathways such as MAPK, AMPK, glucagon, PI3K-Akt, insulin, and actin cytoskeleton regulation.

### 2.8. Validation of Differentially Expressed miRNAs by RT-qPCR

**Figure 9:**
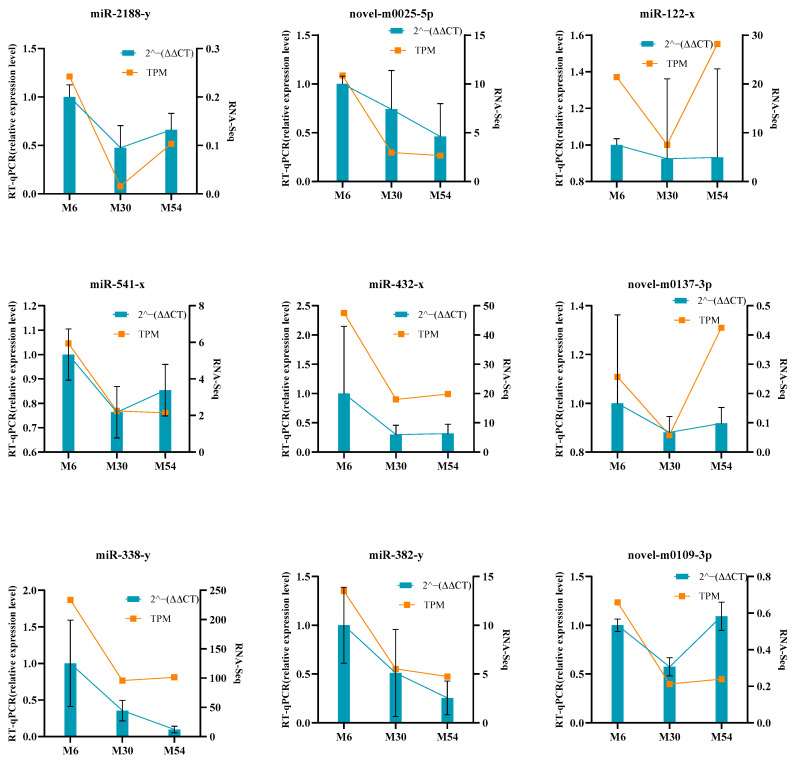
RT-qPCR validation results for small RNA sequencing. RT-qPCR validation was performed on 9 DE miRNAs. These data show the means (means ± SDs) of three replicates. Error bars indicate standard deviations.

We randomly selected nine miRNAs for RT-qPCR validation to verify the DE miRNAs. Comparison of the miRNA expression levels with the RNA-Seq data showed similar patterns (Figure 9), suggesting that the RNA-Seq data were credible.

### 2.9. Construction of the miRNA–mRNA Interaction Network

**Figure 10:**
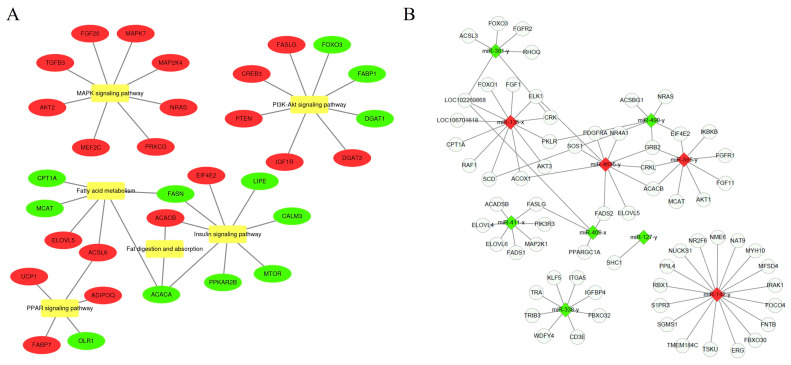
Interaction network of DE miRNAs. (**A**) Network analysis of DE miRNA target genes and their enrichment pathways. Red represents the upregulated mRNAs, green represents the downregulated mRNAs, and yellow represents the enriched signaling pathways. (**B**) Co-expression network of DE miRNAs and its target genes. Red represents the miRNAs whose expression is upregulated, green represents downregulated miRNAs, and blue represents mRNAs.

Background genes associated with signaling pathways related to muscle development and fatty acid metabolism were screened to further understand the DE mRNAs associated with muscle and IMF development (Figure 10A). These genes were then used as target genes to construct miRNA–mRNA interaction networks (Figure 10B). The results showed that the miRNA–mRNA regulatory relationship in this organism was more complex and that one mRNA might be regulated by multiple miRNAs, such as *SCD*, *GRB2*, *ACACB*, *CRK*, *ACOX1*, and so on. Further analysis showed that the upregulated expression pattern had a stronger miRNA–mRNA regulatory relationship than the downregulated expression pattern. In this network, several important relationship axes were screened as miR-885-y-*ACACB*, miR-4695-y-*ACACB*, miR-381-x-*ACSL3*, miR-142-y-*FBXO30*, etc.

## 3. Discussion

Muscle fiber diameter, connective tissue, and intramuscular fat are the main factors affecting meat tenderness. The connective tissue content of muscle is an important factor in meat tenderness, and its main component is collagen fibers. Dransfield et al. found a strong positive correlation between collagen fiber content and muscle shear in beef, indicating that the higher the collagen fiber content, the worse the meat tenderness [25]. Myofibers are the most basic units that make up a muscle. The smaller the diameter and the area of myofibers, the better the muscle tenderness [26]. In the present study, the HE staining results showed that the diameter and area of the myofibers of Tianzhu white yak were smallest at the age of 6 months and tended to increase significantly with age, while the density of myofibers was largest at the age of 6 months and tended to decrease significantly with age. We used histological quantitative analysis to analyze the collagen fiber content of skeletal muscle in Tianzhu white yak during the growth process, which showed a tendency of increase and then decrease, with the lowest collagen fiber content at the age of 6 months, and the difference was highly significant (*p* < 0.05) with respect to the other groups. After the birth of Tianzhu white yak, the yak calf can obtain a lot of nutrients from the breast milk, which can provide a guarantee for the increase in collagen fiber, and the outside living environment after the birth of the fetus is a strong stimulus for the growth and development of the organism. As the calf’s locomotor ability increases, the collagen fiber content increases, which can provide support and connection for the skeletal muscle and coordinate the transmission of force, information, and nutrients to the skeletal muscle, providing a strong help. From 30 to 54 months of age, the collagen fiber content decreased slightly. One possible reason for this is that the collagen content of Tianzhu white yak decreased with age due to the increase in the diameter of skeletal muscle myofibers and the deposition of intermuscular adipose tissue. It can be seen that higher collagen fiber content results in having poorer muscle tenderness, indicating that muscle tenderness is determined by a combination of factors. It has been reported that the greater the cross-sectional area of muscle fibers, the higher the carcass weight of pigs [27]. The above findings suggest that these differences are the key factors affecting meat production performance and meat quality performance in yak of different ages.

IMF is the accumulation of fat-soluble and lipid-like substances in muscle, which is one of the most important indicators for determining meat quality and contributes to muscle tenderness, which in turn affects meat flavor [28]. In this study, the IMF contents of yak of different ages differed significantly (*p* < 0.05), and the IMF contents of 54-month-age and 30-month-age yak were significantly higher than that of 6-month-age yak, being about twice as high. It was shown that IMF content increased significantly (*p* < 0.05) with age in both Gannan yak and Qinchuan yak, with the IMF content in yak less than 3 years old being significantly lower than that in yak 4~7 years old [29].

The lipids in meat are crucial in beef quality evaluation, and the lipid content in muscle can affect many indexes of meat traits [11,30]. Due to its ability to deposit large amounts of intramuscular marbled fat, snowflake beef has a better flavor than regular beef [31]. During growth and development of the animal body, skeletal muscle growth is primarily the result of muscle hypertrophy accompanied by the proliferation of skeletal muscle satellite cells, which integrate new nuclei into existing muscle fibers [32]. During the growth and development of the animal body, the latest tissue to develop is fat, and a large accumulation of fat in muscle leads to the formation of IMF. It has been proved that IMF content and fatty acid composition have a certain influence on meat quality in terms of shear, tenderness, flavor, and sensory score [33]. However, there are fewer studies on the genetic mechanisms of muscle development and IMF deposition in yak. In addition, studies on muscle development and IMF deposition in yak have mainly focused on individual genes or pathways, which do not allow for a comprehensive study of the regulatory mechanisms of muscle development and IMF deposition, whereas the study of related genes and sequencing of yak muscle development and IMF deposition can help to explore the regulatory networks and signaling pathways. Muscle development and IMF deposition are the result of precise regulation of hormones, genes, non-coding RNAs, and other factors. Spatiotemporal expression, transcription and translation, signaling, and feedback mechanisms are their modes of regulation [34,35,36].

MiRNAs are important post-transcriptional molecules regulating adipogenesis and lipid accumulation [37]. A growing body of research suggests that miRNAs play key regulatory roles in the development of tissues across species, for instance, Columba Livia [38], chicken [39], goats [40], people [41], Wuranke sheep [42], cattle [43], pigs [44], and fish [45]. At the same time, more and more studies have shown that miRNAs play critical regulatory roles in the development of various tissues, for instance, skeletal muscle [46], brain [47], mammary [48], adipose [49], heart [50], plant [51], uterus [52], gonadal [53], tooth [54], and hair follicle [55] tissues. MiRNAs related to muscle development and fat deposition have also been reported: those associated with muscle development include miR-1, miR-133, and miR-206 [56], miRNA-454 [57], novel-miR-158 [58], novel-m0036-3p, and novel-m0037-3p [59]; and those associated with fat deposition include bta-miR-142-3p [60], bta-miR-122 [61], miR-424 [62], novel-miR-667, and novel-miR-126 [63]. However, to date, less research has been carried out on the role of miRNAs in muscle development and IMF deposition in Tianzhu white yak. In the present study, we conducted high-throughput RNA-Seq sequencing to identify miRNAs associated with muscle development and fat deposition characteristics in yak. A total of 1191 miRNAs and 22,061 mRNAs were identified, including several previously unreported novel miRNAs. Moreover, we identified 225 DE miRNAs in the longissimus dorsi muscle tissues of Tianzhu white yak at different ages. Previous studies demonstrated that miRNAs regulate the expression of neighboring mRNAs and modulate biological functions [64,65,66,67,68,69]. Therefore, we hypothesized that miRNAs may be key regulators that promote muscle growth and IMF deposition in yak.

MiRNAs, as important post-transcriptional regulatory elements of genes, have been widely reported for their role in regulating lipid metabolism and synthesis [70,71,72,73]. Growing evidence suggests a role for miRNAs in the regulation of muscle growth and development in animals [74,75,76,77]. Gene silencing mediated by miRNAs plays an important role in animal development and disease [78,79,80]. Other studies on skeletal muscle have shown that miRNAs appear to be important during muscle development and the deposition of IMF, both of which can impact final meat quality [39,40,61,62,63,81]. It has been found that miR-381 is an important miRNA associated with intramuscular fat deposition in LWQ female yak; however, it was observed that miR-381 was downregulated in LM and adipose tissue from 0.5 to 2.5 years of age [81]. In this study, small RNA sequencing revealed that miR-381-y was downregulated in the longissimus dorsi muscle tissues of M6, M30, and M54 Tianzhu white yak, which was basically in line with the results of the previous study. Therefore, miR-381-y was hypothesized to be related to intramuscular lipid deposition in the muscles of Tianzhu white yak. We found that muscle-specific miRNAs (miR-29, miR-206, miR-208, miR-130, miR-133, and miR-499) had high expression levels during yak skeletal muscle development, which was similar to what was observed in bovines, mice, patients, and pig [82,83,84,85]. They are also important participants in the normal differentiation and proliferation of myoblasts [86].

The GO annotation illustrated that the predicted target genes of DE miRNAs were mainly classified in the metabolic process of acyl coenzymes, lipid regulation, the coenzyme A biosynthesis process, and the negative regulation of fatty acid metabolism and exerted roles in CoA carboxylase activity, ligase activity, formation of carbon–nitrogen bonds, purine ribonucleoside binding, and cation binding. Interestingly, KEGG pathway annotation of the target genes identified five pathways of interest to us related to adipocyte differentiation and lipid metabolism, namely, the insulin signaling pathway, fat digestion and absorption, fatty acid metabolism, the PPAR signaling pathway, and the MAPK signaling pathway, and one pathway of interest to us related to muscle development, namely, the PI3K-Akt signaling pathway. The insulin signaling pathway is associated with metabolism and growth in animal organisms and is highly conserved during evolution [87]. In the present study, we identified two miRNAs highly expressed in the insulin signaling pathway, miR-4695-y and miR-885-y. The common target DE mRNAs of these two DE miRNAs are acetyl-CoA carboxylase β gene (*ACACB*) and others. Among them, the *ACACB* gene has been found to be involved in fatty acid oxidative metabolism, which regulates the balance of blood glucose and fat metabolism in animal organisms by participating in the metabolic process [88]. A study screening statistical analysis in Japanese Black steer semitendinosus (STD) muscle revealed that miR-196a/b and miR-885 are expressed in STD muscle and are specific genes for fast-type muscle. In addition, the specific distribution of miR-196a/b and miR-885 suggests that these miRNAs target mRNAs that determine the phenotype of slow-type muscles [89]. A study exploring the relationship between body condition at parturition and expression profiles of colostrum miRNAs in dairy cows found that miR-885 was downregulated in cows with elevated circulating FFA [90]. Currently, there are fewer studies on miR-4695, and the studies that do exist focus on disease. In summary, miR-885 and miR-4695 can be the main DE miRNAs screened in this study, and the DE mRNAs targeted by these two DE miRNAs can contribute to subsequent in-depth studies in yak muscle development and IMF deposition.

The MAPK signaling pathway can control biological processes through various cellular mechanisms involving activation/inhibition of related factors [91]. The MAPK signaling pathway promotes the adaptation of skeletal muscle to exercise metabolism in the body and plays an important role in this process [92]. One study identified miR-135 associated with muscle development, with miR-135 targeting myocyte-specific enhancer factor 2C (MEF2C) [93]. *MEF2C* is a member of the myocyte-specific enhancer factor 2 (MEF2) family, which regulates skeletal myogenesis. It is a transcription factor important for myogenin expression and has a synergistic regulatory effect [94]. MEF2 proteins play important roles in many muscle-specific gene regulation as well as differentiation processes [95]. It has been shown that the transcriptional function of MEF2C is essential in mammalian myogenesis [96]. *MEF2C* is essential for bone and skeletal muscle development and plays a regulatory function in cardiovascular and neural cell development [97,98]. LIU et al. found that in mice with muscle damage, after knockout of *MEF2C*, the muscle repair process was significantly inhibited, and the myoblast differentiation process was seriously hindered [99]. *MEF2C* affects Chinese native cattle stature (both size and weight) [100]. Juszczuk-Kubiak et al. identified *MEF2C* as having the potential to influence beef quality in different cattle breeds [101]. Zhao et al. showed that *MEF2C* mRNA stability promotes the differentiation of skeletal muscle satellite cells in goats [102]. Li et al. showed that *MEF2C* promoted growth traits such as body weight in Nanjiang yellow goats [103]. Ren et al. showed that *MSTN* can regulate fatty acid metabolism through the MEF2C–miR222–SCD5 cascade reaction, which in turn modulates the effects of fat deposition [104]. In this study, we identified *MEF2C*, a key target gene in the MAPK signaling pathway, and we also identified miR-135, which is associated with muscle development, and the above findings can be used to support the results of this study. Therefore, we hypothesize that *MEF2C* and miR-135 can be used as key signals in the MAPK signaling pathway screened for association with muscle development and IMF deposition.

Trend analysis can provide further analysis of muscle development at different developmental stages, thus revealing specific patterns during age changes. Therefore, we performed trend clustering analysis for all DE miRNAs. As a result, it was found that Profile 1 and Profile 2 were in the expression downregulation mode, containing 54 and 33 DE miRNAs, respectively, with the highest number of DE miRNAs in Profile 1. Furthermore, in addition, several pairs of miRNA–mRNA targeting relationships of interest associated with muscle development and intramuscular fat deposition were screened, such as miR-381-y-*FOXO3*, miR-381-y-*FOXO3*, miR-4695-y-*ACACB*, miR-885-y-*ACACB*, miR-4695-y-*ELOVL5*, miR-142-y-*FBXO30*, miR-338-y-*KLF5*, and miR-338-y-*FBXO32*. Therefore, we hypothesized that the consistently downregulated miRNAs may play a more important regulatory role during muscle growth and IMF deposition in the Tianzhu white yak.

## 4. Materials and Methods

### 4.1. Animals and Sample Collection

In total, 18 male Tianzhu white yak (6 each in age groups of 0.5, 2.5, and 4.5 years) reared on natural pasture were selected from farmers’ households in Tianzhu County, Gansu Province, China. All yak grazed in a purely natural way without supplemental feeding and in approximately the same state of health. All yak were fasted for 24 h before slaughter. After slaughter, the longissimus dorsi muscle tissue of each yak was collected and placed in liquid nitrogen, then brought back to the laboratory for RNA-Seq and RT-qPCR. Total RNA was extracted using TRIzol reagent (Invitrogen Corporation, Carlsbad, CA, USA) according to the manufacturer’s instructions. The RNA was then treated with DNase I (Takara, Dalian, China) to remove genomic DNA. The quality and integrity of RNA were verified by electrophoresis, using a NanoDrop^TM^ 2000 Spectrophotometer (Thermo Fisher Scientific, Waltham, MA, USA) and an Agilent 2100 bio-analyzer (Agilent Technologies, Palo Alto, CA, USA). RNA Integrity Numbers (RINs) ≥ 6.5 and 28S/18S ratios ≥ 1.0, were used to construct the sequencing library.

A 1 cm^3^ muscle sample from the same site was collected and preserved in 4% neutral paraformaldehyde for muscle histological characterization. Approximately 0.5 kg of longissimus dorsi muscle samples were collected and placed in a 4 °C incubator for the determination of IMF contents.

### 4.2. Analysis of the Intramuscular Fat Content

The IMF contents of the 9 longissimus dorsi samples (3 yak for each age group) were determined according to the standard Soxhlet extraction method [105]. In brief, the samples were pre-dried and crushed following the weighing of an x amount (in grams) into the Soxhlet glass tube and then transferred to the extraction chamber in the Soxhlet equipment. Each sample was soaked overnight in anhydrous ether, following which the anhydrous ether backflow devices were opened for 10 h at 80 °C. The residue sample was dried under a fume hood for 1 h and then transferred to a forced-air oven at 105 °C for 8 h. The dried residue sample was weighed and marked as the y amount (in grams). The IMF content was calculated as follows:

$$IMF\left(\%\right)=\frac{x-y}{x}\times 100\%$$

Statistical analysis was performed using SPSS 26.0 software, and the general linear model was used for the test of significance, with the results expressed as means ± standard deviations (means ± SDs), with *p* < 0.05 being significant.

### 4.3. Staining of Muscle Tissue Using Hematoxylin and Eosin and Masson Staining of Collagen Fibers

Muscle tissues were fixed in 4% neutral paraformaldehyde immediately after collection, and paraffin sections were prepared after 48 h of fixation. The whole process of staining included embedding, sectioning, staining, dehydration, sealing, and image acquisition. First, paraffin sections were deparaffinized. Next, the sections were put into Bouin liquid at room temperature overnight and then rinsed with running water until the yellow color on the sections disappeared. Then, the sections were stained with azurite blue staining solution for 2~3 min and washed with water, after which they were stained with Mayer hematoxylin staining solution for 2~3 min and washed with water. The sections were differentiated with acidic ethanol differentiation solution for a few seconds and rinsed with running water for 10 min, then stained with Lichtenstein’s Red Magenta Staining Solution for 10 min and rinsed with distilled water. The sections were then treated with phosphomolybdic acid solution for about 10 min, after which the upper solution was poured off, and the sections were stained directly with aniline blue staining solution for 5 min without washing in water, followed by rinsing with 1% acetic acid solution for 2 min, rapid dehydration with 95% ethanol, then dehydration with anhydrous ethanol 3 times, each time for 5–10 s. Next, the sections were transparentized with xylene 3 times, each time for 1–2 min, Finally, the slices were sealed with neutral resin, and the results of the collagen fiber staining in muscle tissue were observed under an IX73 microscope (Olympus, Tokyo, Japan) and the area to be measured was selected and photographed at 400× for measurement. The method described by Zhang [106] was used to carry out the HE staining.

CaseViewer2.2 (3 DHISTECH Ltd. Budapest, Hungary) scanning browser software was utilized to select target regions for data analysis, with eight regions per slice. The diameter, cross-sectional area, and density of muscle fibers after HE staining were measured using the image analysis software Motic Images Advanced 3.2. The optical density and area of Masson-stained images were determined using the Image-Pro Plus 6.0 image analysis system [107], and the percentage of collagen fiber area was calculated. An independent-samples *t*-test was performed on the measurement results using SPSS v26.0 software, and the data were expressed as means ± standard errors.

### 4.4. RNA Extraction, Library Construction, and Sequencing

The RNA of the longissimus dorsi muscle tissue of Tianzhu white yak was extracted by the Trizol method (Invitrogen, Carlsbad, CA, USA). The concentration of total RNA was assessed using a Nanodrop 2000 (Thermo Scientific, Waltham, MA, USA). The integrity of the total RNA was determined according to the 2100 Bioanalyzer (Agilent, Palo Alto, CA, USA) assay kit. RNA samples with RNA Integrity Numbers (RINs) > 7.5 were used to construct cDNA libraries using TruSeq^TM^ Small RNA Sample Prep Kits (Illumina, San Diego, CA, USA), and subsequently sequenced on the Illumina Novaseq6000 (Illumina, San Diego, CA, USA) platform by Gene Denovo Biotech Co., Ltd. (Guangzhou, China).

### 4.5. Sequence Analysis and Characterization of miRNAs

The raw data were subjected to quality control using fastp (version 0.18.0), including removal of splice sequences, sequences without insert fragments, sequences with insert fragments less than 18 nt in length, low-quality base sequences at the end of a sequence, and sequences containing polyA (where more than 70% of the bases in a sequence were A). The total number, type, and length distribution of sequences were then calculated, and final small RNA sequence data (clean data) were obtained for subsequent analysis. Clean and high-quality reads 16–35 nt in length were used in subsequent analyses. o determine the distribution of miRNAs based on the reference sequences, high-quality clean reads were aligned with the yak reference genome sequence (ncbi_GCF_000298355.1) using TopHat2 software (BosGruv2.0, NCBI) to obtain positional information on the reference genome or genome to obtain mapped reads. Tag sequences that mapped to exons or introns were removed, as these fragments could have been produced by mRNA degradation. The remaining high-quality clean reads were then aligned to the miRbase v22.0 database to annotate known yak miRNAs and highly conserved miRNAs in other species. Finally, high-quality clean read sequences that were not aligned to the miRbase v22.0 database but were aligned to the yak reference genome were predicted using miReap v0.2 software. Read sequences were predicted, and new candidate miRNAs were identified. Miranda (v3.3a) and Target Scan (v7.0) software were used to predict the target genes of DE miRNAs in the longissimus dorsi muscle tissues of the Tianzhu white yak from M6, M30, and M54.

### 4.6. Differential Expression Analysis and Time-Series Expression Profile Clustering

Total miRNA consists of known miRNAs and novel miRNAs, based on different libraries constructed from longissimus dorsi muscle tissues. The miRNA expression levels were calculated and normalized to transcripts per million (TPM). DE miRNAs were identified using the negative binomial generalized linear model implemented in DESeq2 software (V1.20.0), with |fold change| > 2 and *p*-value < 0.05 [108]. In addition, older yak were used as controls and compared with each other. To cluster and visualize the profiles of the DE miRNAs over the three age groups, they were analyzed by Short Time-series Expression Miner [109] software (Ernst and Bar-Joseph, 2006). Finally, they were analyzed and screened for biologically significant expression patterns.

### 4.7. Prediction of miRNA Targets, GO Enrichment, KEGG Pathway Analysis, and Integrated Analysis of miRNAs and Their Target Genes

For DE miRNAs, potential target genes of DE miRNAs were obtained based on Target Scan 8.0 [110] and miRanda 3.3 [111]. The intersection of the results was more likely to be chosen as the predicted miRNA target genes. These target genes corresponded to the mRNA transcriptome profile. GO (Gene Ontology, GO) biological function enrichment analysis and KEGG (Kyoto Encyclopedia of Genes and Genomes, KEGG) signaling pathway enrichment analysis of genes were conducted on the target genes of miRNAs by GOATOOLS [112] and KOBAS 2.0 [113], respectively.

### 4.8. Analysis of miRNA–mRNA Regulatory Networks

In order to further explore the molecular mechanisms by which DE miRNAs regulate intramuscular fat development in yak, the most significantly upregulated and the most significantly downregulated miRNAs were selected and combined with the results of the target gene prediction, and the miRNA–mRNA regulatory network was constructed using Cytoscape v3.7.1 software [107].

### 4.9. RT-qPCR

To verify the accuracy of RNA-Seq, 9 DE miRNAs were randomly selected in this study and quantified using RT-qPCR. The total RNA of the sequenced backmost longissimus dorsi muscle tissue was first reverse-transcribed into cDNA using the miRNA First Strand cDNA Synthesis Kit (Accurate Biology, Changsha, China) and then subjected to real-time fluorescence quantitative PCR. Reverse transcription reaction system: total RNA, *TransScript*^®^ miRNA RT Enzyme Mix, 2 × TS miRNA Reaction Mix, and RNase-free water to the PCR tube to form a 20 µL reaction system. Reverse transcription reaction conditions were as follows: they were mixed and incubated at 37 °C for 1 h and heated at 85 °C for 5 s to inactivate RT Enzyme Mix, synthesize miRNA first-strand cDNA. The RT-qPCR reaction system was prepared according to the recommended reaction system (20 μL) of the Tigen Fluorescence Quantification Kit (Tiangen Biotech, Beijing, China) (Table 3). The reaction conditions were as follows: pre-denaturation at 95 °C for 5 min; denaturation at 95 °C for 20 s; annealing at 55 °C for 20 s; extension at 72 °C for 20 s. The reaction was carried out for 40 cycles, and the solubilization curve was prepared at 65 °C~95 °C. The reaction was performed on an Applied Biosystems QuantStudioR 6 Flex real-time fluorescence quantitative PCR instrument, with 3 replicates for each sample (Thermo Lifetech, Waltham, CA, USA).

The selected DE miRNAs and RT-qPCR primer sequences are shown in Table 4, and the downstream primers for miRNAs were used with special primers that came with the kit. The *U6* snRNA was used as an endogenous control for miRNAs, and the primers were synthesized by TsingKe Biotechnology Co., Ltd. (Xi’an, China). All the reactions were repeated in triplicate, and the relative expression levels were calculated using the 2^−ΔΔct^ method.

## 5. Conclusions

In summary, we have reported the expression characteristics of miRNAs in the longissimus dorsi muscle of Tianzhu white yaks for the first time. A total of 225 DE miRNAs and 450 DE mRNAs were identified in the cDNA libraries of eighteen Tianzhu white yaks. The analysis revealed that the target genes of DE miRNAs overlapped with some DE mRNAs and screened for target genes associated with muscle development (*IGF1R*, *CREB3*, *FOXO3*, *FABP1*, *FASLG*, and *PTEN*) and IMF deposition (*ACACA*, *ACACB*, *UCP1*, *OLR1*, *ADIPOQ*, *ACSL6*, *ELOVL5*, *FASN*, and *PPKAR2B*). Characterization of the three groups of these target genes suggested that miRNAs may play an important regulatory role in muscle growth and development in Tianzhu white yak. Our results provide new candidate molecular markers for the selection and improvement of yak and provide a theoretical basis for studying the molecular mechanisms of key non-coding RNAs in intramuscular pre-adipocyte differentiation.

## Figures and Tables

**Table 1:** Overview of small RNA sequencing.

Sample	Average Clean_Reads	Average High_Quality	Average 3′Adapter_Null	Average Insert_Null	Average 5′Adapter_Contaminants	Average polyA	Average Clean_ Tags
M6	13,786,039 (100%)	13,669,782 (99.1560%)	12,780 (0.0911%)	53,070 (0.3819%)	6541 (0.0467%)	173 (0.0012%)	13,227,222 (96.1951%)
M30	16,023,867 (100%)	15,874,203 (99.1314%)	10,477 (0.0579%)	51,679 (0.3151%)	5659 (0.0420%)	209 (0.0012%)	15,627,938 (96.7044%)
M54	16,575,445 (100%)	13,640,341 (98.9557%)	14,968 (0.0780%)	85,117 (0.4558%)	9939 (0.0580%)	284 (0.0015%)	16,518,724 (96.4666%)

**Table 2:** Clean reads and reference genome alignment results.

Sample	Average Total_Abundance	Average Match_Abundance	Average Other_Abundance
M6	13,517,788	8,987,399 (75.75%)	3,451,495 (23.93%)
M30	15,500,216	12,281,680 (79.21%)	3,467,715 (20.79%)
M54	15,990,108	11,910,843 (78.73%)	3,335,252 (21.27%)

**Table 3:** RT-qPCR reaction system.

Reagent	Usage Amount
2 × SuperReal premix Plus	10 μL
Upstream primer	0.4 μL
Downstream primer	0.4 μL
cDNA templates	2 μL
50 × ROX Reference Dye1	0.4 μL
RNase-Free ddH_2_O	0.8 μL

**Table 4:** Primer information for the validation of DE miRNAs using RT-qPCR.

miRNA/Gene	Forward Primer	Reverse Primer
miR-2188-y	GCTGTGTGAGGTCGGACCTATC	
novel-m0025-5p	AGGACTCCATTTGTTTTGATGA	/
miR-122-x	TGGAGTGTGACAATGGTGTTT	/
miR-541-x	AAAGGATTCTGCTGTCGGTCCCACT	/
miR-432-x	TCTTGGAGTAGGTCATTGGGTGT	/
novel-m0137-3p	CCGAGCCTGACAGATCACACAC	/
miR-338-y	TCCAGCATCAGTGATTTTGTT	/
miR-382-y	AATCATTCACGGACAACACTTT	/
novel-m0109-3p	AGCCCGAGGGTCCCAAGCTGAGC	/
*U6*	ACGGACAGGATTGACAGATT	TCGCTCCACCAACTAAGA

## Data Availability

The data presented in the study are deposited in the NCBI repository, https://www.ncbi.nlm.nih.gov/sra/PRJNA1098498, (accessed on 17 April 2024), accession number PRJNA1098498.

## Supplementary Materials

The supporting information can be downloaded at: https://www.mdpi.com/article/10.3390/ijms251810151/s1.
